# Finasteride Concentrations and Prostate Cancer Risk: Results from the Prostate Cancer Prevention Trial

**DOI:** 10.1371/journal.pone.0126672

**Published:** 2015-05-08

**Authors:** Cindy H. Chau, Douglas K. Price, Cathee Till, Phyllis J. Goodman, Xiaohong Chen, Robin J. Leach, Teresa L. Johnson-Pais, Ann W. Hsing, Ashraful Hoque, Catherine M. Tangen, Lisa Chu, Howard L. Parnes, Jeannette M. Schenk, Juergen K. V. Reichardt, Ian M. Thompson, William D. Figg

**Affiliations:** 1 Genitourinary Malignancies Branch, Center for Cancer Research, National Cancer Institute, Bethesda, Maryland, United States of America; 2 Swog Statistical Center, Fred Hutchinson Cancer Research Center, Seattle, Washington, United States of America; 3 Department of Urology, University of Texas Health Science Center at San Antonio, San Antonio, Texas, United States of America; 4 Cancer Prevention Institute of California, Fremont, California, Stanford Cancer Institute, Palo Alto, California, United States of America; 5 Department of Clinical Cancer Prevention, The University of Texas M.D. Anderson Cancer Center, Houston, Texas, United States of America; 6 Division of Cancer Prevention, National Cancer Institute, Bethesda, Maryland, United States of America; 7 Cancer Prevention Program, Fred Hutchinson Cancer Research Center, Seattle, Washington, United States of America; 8 School of Pharmacy and Molecular Sciences, James Cook University, Townsville, Queensland, Australia; Innsbruck Medical University, AUSTRIA

## Abstract

**Objective:**

In the Prostate Cancer Prevention Trial (PCPT), finasteride reduced the risk of prostate cancer by 25%, even though high-grade prostate cancer was more common in the finasteride group. However, it remains to be determined whether finasteride concentrations may affect prostate cancer risk. In this study, we examined the association between serum finasteride concentrations and the risk of prostate cancer in the treatment arm of the PCPT and determined factors involved in modifying drug concentrations.

**Methods:**

Data for this nested case-control study are from the PCPT. Cases were drawn from men with biopsy-proven prostate cancer and matched controls. Finasteride concentrations were measured using a liquid chromatography-mass spectrometry validated assay. The association of serum finasteride concentrations with prostate cancer risk was determined by logistic regression. We also examine whether polymorphisms in the enzyme target and metabolism genes of finasteride are related to drug concentrations using linear regression.

**Results and Conclusions:**

Among men with detectable finasteride concentrations, there was no association between finasteride concentrations and prostate cancer risk, low-grade or high-grade, when finasteride concentration was analyzed as a continuous variable or categorized by cutoff points. Since there was no concentration-dependent effect on prostate cancer, any exposure to finasteride intake may reduce prostate cancer risk. Of the twenty-seven SNPs assessed in the enzyme target and metabolism pathway, five SNPs in two genes, *CYP3A4* (rs2242480; rs4646437; rs4986910), and *CYP3A5* (rs15524; rs776746) were significantly associated with modifying finasteride concentrations. These results suggest that finasteride exposure may reduce prostate cancer risk and finasteride concentrations are affected by genetic variations in genes responsible for altering its metabolism pathway.

**Trial Registration:**

ClinicalTrials.gov NCT00288106

## Introduction

Prostate tissue growth and differentiation is dependent on androgen hormones and regulated via the androgen receptor (AR) [1,2]. Testosterone is irreversibly converted to the more physiologically potent androgen 5α-dihydrotestosterone (DHT) mediated by the androgen metabolizing enzymes steroid 5a-reductase types I and II in the prostate tissue (encoded by the *SRD5A1* and *SRD5A2* genes, respectively) [3] and also by steroid 5α-reductase type III (encoded by *SRD5A2L* or *SRD5A3*) recently identified in castration-resistant prostate cancer cells [4,5].

Finasteride is a specific and potent SRD5A2 inhibitor [6,7,8], but it can also block the SRD5A1 enzyme, although at a much slower rate [9] and SRD5A3 activity [10]. Several mutations on the *SRD5A* genes have been reported that changed the expression level of 5α-reductase [11,12]; thus genetic polymorphisms in these genes may affect enzyme activity and thus lead to individual variability in drug efficacy. Finasteride is extensively metabolized in the liver, primarily via the *CYP3A* subfamily, involving CYP3A4-mediated hydroxylation and oxidation reactions [13]. The *CYP3A* subfamily exhibits high sequence homology and various isoforms (e.g., CYP3A4 and CYP3A5) share similar substrate specificity. In fact, CYP3A5 is reported to be a major contributor to the metabolism of many CYP3A-mediated drugs and is highly polymorphic [14]. There is currently no published literature on the effects of *CYP3A4* and *CYP3A5* genetic variations and finasteride metabolism.

Treatment with finasteride results in a significant reduction of prostatic and circulating DHT levels. Due to the role of DHT in the development of prostate cancer, it was hypothesized that finasteride could be used effectively as a chemopreventive agent to reduce the risk of this disease. In the Prostate Cancer Prevention Trial (PCPT), a randomized, placebo-controlled trial testing whether finasteride could reduce the 7-year period prevalence of prostate cancer, finasteride reduced the risk of prostate cancer by 25%, even though high-grade prostate cancer was more common in the finasteride group [15].

Here, we investigate the association of serum finasteride concentrations with prostate cancer risk using a nested case-control study in the finasteride-treated arm of the PCPT. We also examine whether polymorphisms in the enzyme target and metabolism genes of finasteride, such as *SRD5A2*, *CYP3A4*, and *CYP3A5*, are related to drug concentrations. Findings from this study could provide further insight into the role of finasteride for cancer prevention and the etiology of the increased risk of high-grade cancer among men treated with finasteride, as well as improve our understanding of the interindividual responses to finasteride treatment.

## Methods

### Study Design, Study Population, and Data Collection

All data for this study are from the PCPT (SWOG-9217) [15,16]. Details of the study design and participant characteristics have been described previously [15,16]. Briefly, 18,882 men age 55 years and older with a normal digital rectal exam (DRE), prostate specific antigen (PSA) level of 3 ng/mL or below, and no history of prostate cancer or other clinically significant co-morbid conditions that would have precluded successful completion of the study protocol, were randomized to receive either finasteride (5 mg/day) or placebo daily for seven years. During the course of the PCPT, men underwent annual DRE and PSA measures and a prostate biopsy was recommended for all men with an abnormal DRE or a finasteride-adjusted PSA of > 4.0 ng/mL. At the conclusion of the trial, either a prostate cancer diagnosis or end-of-study biopsy was available from 59.6% of the participants in the finasteride treatment arm, and 63% from the placebo arm. This level of ascertainment agreed well with the study design assumption that 60% of men would have an endpoint assessed. All men signed informed consent and study procedures were approved by the Institutional Review Boards of the participating 221 study sites [15,16].

This study reported here is part of a large nested case-control study designed to examine multiple hypotheses about prostate cancer and risk. Cancer cases and controls in this report were from the finasteride-treated study arm. Cases were men with biopsy-determined prostate cancer identified either by a for-cause or end-of-study biopsy and who had DNA from white blood cells or serum available. Controls were selected from men who completed the end-of-study biopsy procedure, had no evidence of prostate cancer and had archived DNA samples. Controls were frequency matched to cases on distributions of age (in 5-year age groups), and positive family history for first degree relative with prostate cancer. Controls were oversampled on race to include all eligible non-white subjects to maximize power for subgroup analyses. Finasteride concentrations were available from 749 cases and 758 controls. For this analysis, all men who were non-compliant to study drug were removed. Non-compliance was defined in two ways: 1) finasteride concentrations below the lowest detectable limit of 1 ng/mL (n = 228) and 2) self-report of going off study drug at some point before the finasteride concentration was assessed (additional n = 6). The final sample size for analyses is 597 cases and 676 controls. Of these, 532 cases and 646 controls also had single nucleotide polymorphism (SNP) data available. Participants who were excluded due to lack of adequate DNA were comparable to participants with adequate DNA in terms of demographic characteristics such as age, BMI, race, and family history (data not shown).

Details regarding age, race/ethnicity, family history, physical activity (type, frequency, duration, pace, and intensity), usual alcohol consumption and history of smoking were collected at baseline using self-administered questionnaires. Clinic staff measured height and weight at randomization, and body mass index (BMI) was calculated as weight (kg) divided by height^2^ (m). Tumors were graded centrally and categorized as low grade = Gleason < 7; high grade = Gleason ≥ 7, retaining the definitions used in the original trial report.

### Blood Collection and Genotyping

Non-fasting blood specimens were collected at screening and yearly thereafter. Venous blood was drawn into collection tubes without anticoagulant and serum was centrifuged, aliquoted, and stored at -70°C until analysis. DNA was extracted from white blood cells using Qiagen M48 robot (Valencia, CA) at NCI Frederick and then shipped to the Roswell Park Cancer Institute Genomics Core Facility and the University of Texas Health Science Center at San Antonio for genotyping by polymerase chain reaction (PCR) amplification using the Sequenom, Taqman, or Illumina platform assays. Briefly, SNP genotypes were determined using the Illumina VeraCode GoldenGate genotyping assay (Illumina Inc.; San Diego, CA). The list of SNPs were submitted to Illumina and scored with the Assay Design Tool (ADT). Those SNPs with acceptable scores were developed into an oligonucleotide pool assay (OPA) designed for a VeraCode GoldenGate panel. Two hundred fifty nanograms of DNA were used as the template for the assay. The assay was performed in 96 well plates following the established protocol (Illumina). The plates were scanned using an Illumina BeadXpress reader and the genotypes were analyzed using GenomeStudio software (Illumina). Interplate and intraplate replicates were included as quality control measures. Twenty-seven SNPs in *SRD5A2*, *SRD5A2L*, *CYP3A4*, and *CYP3A5* were genotyped. Primer sequences will be provided upon request.

### Sample Collection and Measurement of Finasteride Serum Concentrations

For most men (90%), samples used to determine finasteride concentrations were measured at 3 years post-baseline. For the other 10%, time points ranged from 1–7 years post-baseline. Steady state finasteride concentrations were measured using a liquid chromatography-mass spectrometry validated method on a HP 1100 system (Agilent Technology, Palo Alto, CA, USA) coupled with a single-quadrupole mass spectrometric detector (Agilent 1100 MSD), as described previously [17], which was further modified and validated in serum. The lower limit of quantitation for finasteride was established at 1 ng/mL. Laboratory personnel were blinded to the case-control status of all participants. Two sets of QC samples, 20 in each set, were included for quality control, and the coefficients of variation were 6.5% and 7.4%.

### Statistical Analysis

We compared baseline demographic and lifestyle characteristics of prostate cancer cases and controls by student *t* test for continuous variables and chi-square test for categorical variables. Serum concentrations of finasteride were categorized based on clinically defined cut points. Logistic regression was used to calculate ORs and 95% CIs for risk of total prostate cancer, and polytomous logistic regression was used to calculate ORs and 95% CIs of both low-grade and high-grade prostate cancers. The polytomous regression with a generalized logit link permits a model including both low-grade and high-grade cancers as outcomes in the same model, contrasted with no cancer. Tests for linear trend for finasteride concentration were based on an ordinal variable corresponding to rank (lowest to highest). Model covariates were carefully selected based on a priori information about potential confounding as well as diagnostic procedures completed as part of our modeling exercises. Final covariates included age, race (white/black/others), time of day of finasteride blood draw, and family history of prostate cancer. To determine the association between single SNP and finasteride levels among whites, mean concentrations of finsteride were calculated for each allele, and p-values were calculated using linear regression adjusted for age and alcohol consumption. All statistical tests were two-sided, with P < 0.05 considered statistically significant. SAS (version 9.2) and R (version 2.15.1) were used for all statistical analyses. Haploview v4.1 was used for assessing LD patterns and haplotype association statistics [18]. Haplotype blocks were determined using the algorithm of Gabriel et al [19].

## Results

Demographic and lifestyle characteristics of the PCPT study population in the finasteride treatment arm are listed in Table 1. Cases and controls did not differ with respect to age, BMI, physical activity, current smoking status or family history of prostate cancer. Because minorities were oversampled (all eligible non-Caucasians were included) in the control group, there were more blacks or other race (non-whites) in the control group. We examined the potential predictors of finasteride concentrations and found that drug serum concentrations were significantly associated with age at baseline and alcohol consumption specifically more than 30 g/d (Table 2). Mean finasteride concentrations were higher in older participants (p<0.0001) and in individuals who consumed more than 30g/d of alcohol (p<0.02).

**Table 1:** Demographics and lifestyle characteristics of cases and controls of the Prostate Cancer Prevention Trial participants in the treatment arm (n = 1273).

	Control (n = 676)	Case (n = 597)	p-value
	Mean (SD)		
Age (y)	63.8 (5.6)	64.1 (5.8)	NA*
BMI (kg/m^2^)	27.7 (4.1)	27.5 (3.8)	0.54
Waist circumference, cm	102.0 (11.1)	101.8 (10.3)	0.72
Waist:hip ratio	1.0 (0.1)	1.0 (0.1)	0.54
Alcohol intake, g/d	9.1 (13.9)	10.1 (15.1)	0.23
Smoking, pack-years	14.9 (16.8)	14.3 (16.2)	0.58
Finasteride concentrations, ng/mL	30.05 (19.72)	29.35 (19.35)	0.53
	n (%)		
Diabetes	49 (7.2)	30 (5.0)	0.10
Family history of prostate cancer	147 (21.7)	135 (22.6)	NA*
Race			NA*
White (Non-Hispanic)	503 (74.4)	556 (93.1)	
Black	91 (13.5)	27 (4.5)	
Other	82 (12.1)	14 (2.3)	
BMI (kg/m^2^)			0.96
Normal (<25)	181 (27.0)	156 (26.4)	
Overweight (25 to <30)	333 (49.6)	298 (50.3)	
Obese (> = 30)	157 (23.4)	138 (23.3)	
Physical Activity			0.52
Sedentary	130 (19.3)	117 (19.7)	
Light	263 (39.1)	249 (41.8)	
Moderate	213 (31.7)	183 (30.8)	
Active	66 (9.8)	46 (7.7)	
Alcohol Intake			0.71
Nondrinker	159 (23.5)	141 (23.6)	
<30 g/d	459 (67.9)	397 (66.5)	
> = 30 g/d	58 (8.6)	59 (9.9)	
Smoking Status			0.21
Never Smoker	237 (35.1)	204 (34.2)	
Current Smoker	57 (8.4)	36 (6.0)	
Past Smoker	382 (56.5)	357 (59.8)	
Education			0.02
High school or less	145 (21.5)	106 (17.8)	
Some college	210 (31.1)	161 (27.0)	
Graduate/professional school	320 (47.4)	330 (55.3)	

* By study design, controls were frequency matched to cases based on age and family history, and oversampled to include all non-whites.

**Table 2:** Mean finasteride levels (ng/mL) stratified by categories of demographic measures among treatment-compliant men in the Prostate Cancer Prevention Trial finasteride arm.

Characteristic	N	Mean (SD)	p-value
Age *			<0.0001
55–59	322	26.68 (16.58)	
60–64	396	28.23 (18.64)	
65–69	325	31.94 (21.06)	
70+	230	33.40 (21.74)	
Diabetes **			0.56
No	1194	29.64 (19.47)	
Yes	79	30.96 (20.72)	
Family History **			0.40
No	991	29.97 (20.06)	
Yes	282	28.85 (17.62)	
Race **			0.08
Non-hispanic White	1059	29.71 (19.46)	
Black	118	27.05 (18.10)	
Other	96	33.07 (21.75)	
BMI *			0.16
Normal (BMI <25)	337	30.22 (21.47)	
Overweight (BMI 25 to <30)	631	30.22 (19.45)	
Obese (BMI > = 30)	295	27.95 (17.22)	
Physical Activity *			0.76
Sedentary	247	29.85 (20.35)	
Light	512	29.91 (19.98)	
Moderate/Active	508	29.48 (18.68)	
Alcohol Intake *			0.02
Non-drinker	300	28.76 (19.58)	
>0 to <30 g/day Alcohol	856	29.34 (19.09)	
> = 30 g/day Alcohol	117	34.96 (21.99)	
Current Smoking Status **			0.11
Current Non-Smoker	1180	29.48 (19.40)	
Current Smoke	93	32.81 (21.12)	

* p-value is based on a trend test

** p-value is based on an analysis of variance F-test

Table 3 gives the results for the associations of finasteride serum concentrations with prostate cancer risk. Among individuals with detectable finasteride concentrations, there was no association between finasteride concentrations and risk of overall (OR, 0.97; 95% CI, 0.91–1.04), low-grade (OR, 0.96; 95% CI, 0.89–1.04), or high-grade (OR, 1.00; 95% CI, 0.93–1.09) prostate cancer when finasteride concentration was analyzed as a continuous variable or categorized by cutoff points. The results remain the same with high-grade disease classified as Gleason 8–10 (data not shown). Since the odds ratio for prostate cancer based on dose were comparable, we can presume that on average all plasma concentrations have a 25% risk reduction of prostate cancer as was reported in the primary treatment report [15]. Thus, while there was no concentration-dependent effect on prostate cancer, we concluded that any exposure to finasteride intake may reduce prostate cancer risk. A sensitivity analysis was performed for cases diagnosed by for-cause biopsy and the results were similar to Table 3 (data not shown). We found no association between finasteride concentration-dependent effects and prostate cancer risk for cases diagnosed by for-cause biopsy.

**Table 3:** Associations of finasteride concentrations with prostate cancer risk in PCPT, overall and stratified by cancer grade.

		All Cancer		(Low) Gleason 2–6		(High) Gleason 7–10	
Finasteride Concentrations	N control	N case	OR (95% CI)	N case	OR (95% CI)	N case	OR (95% CI)
1 to <10 ng/mL	91	87	ref	51	ref	31	ref
10 to <20 ng/mL	154	123	0.76 (0.51–1.12)	73	0.78 (0.49–1.23)	44	0.74 (0.43–1.28)
20 to <30 ng/mL	128	136	1.09 (0.73–1.62)	81	1.13 (0.72–1.79)	51	1.11 (0.65–1.89)
30 to <40 ng/mL	123	99	0.74 (0.49–1.12)	60	0.78 (0.48–1.26)	35	0.72 (0.41–1.28)
40 to <50 ng/mL	84	72	0.81 (0.52–1.28)	32	0.63 (0.36–1.11)	36	1.13 (0.63–2.03)
50+	95	76	0.82 (0.52–1.29)	41	0.79 (0.46–1.34)	32	0.95 (0.52–1.73)
p-trend			0.42		0.22		0.73
Continuous, per 10ng/mL	675	593	0.97 (0.91–1.04)	338	0.96 (0.89–1.04)	229	1.00 (0.93–1.09)

Odds ratios are adjusted for age, race (white vs black vs others), time of day of blood draw, and family history of prostate cancer.

The association between finasteride concentrations and polymorphisms in enzyme target and metabolism genes of finasteride are shown in Table 4. Of the twenty-seven SNPs assessed, five SNPs in *CYP3A4* (rs2242480; rs4646437; rs4986910) and *CYP3A5* (rs776746; rs15524) were associated with finasteride concentrations. For three of the SNPs, the variant allele was associated with higher finasteride concentrations (e.g. *CYP3A4* rs4986910, P_trend_ = 0.005; *CYP3A5* rs776746 and rs15524, P_trend_ = 0.001 and P_trend_ = 0.006, respectively). The variant allele increased mean finasteride levels by >1.5-fold. The remaining two SNPs were associated with lower finasteride concentrations (e.g. *CYP3A4* rs2242480, P_trend_ = 0.004; and rs4646437, P_trend_ = 0.002) and the homozygous variant alleles reduced mean finasteride levels by one half. A sensitivity analysis for the SNP-finasteride association among controls only was performed and showed statistical significance for only two gene variants, *CYP3A4* rs4646437 (p = 0.04) and *CYP3A5* rs776746 (p = 0.03) (data not shown). These two variants were also statistically significant when cases and controls were combined as shown in Table 4. We found no associations between finasteride concentrations and polymorphisms in enzyme targets of the *SRD5A2* or *SRD5A2L/SRD5A3* genes.

**Table 4:** Association between finasteride concentrations and genes in metabolism and target pathways among white men. ^#^

Gene	SNP	Genotype	N	% Frequency	Finasteride (ng/mL)	p-trend *
					Mean (SD)	
*CYP3A4*	rs2242480	CC	823	85.6	30.30 (19.53)	0.004
		CT	130	13.5	26.21 (17.09)	
		TT	8	0.8	15.07 (8.00)	
	rs2740574	AA	912	93.7	29.90 (19.51)	0.060
		AG	59	6.1	26.45 (14.65)	
		GG	2	0.2	13.62 (15.81)	
	rs4646437	CC	801	83.3	30.35 (19.69)	0.002
		CT	149	15.5	26.48 (17.18)	
		TT	12	1.2	16.85 (7.46)	
	rs4986907	GG	973	100.0	29.62 (19.24)	NA
	rs4986910	AA	953	98.6	29.46 (19.21)	0.005
	(M445T)	AG	14	1.4	43.28 (20.30)	
*CYP3A5*	rs15524	CC	6	0.6	17.17 (8.70)	0.006
		CT	121	12.5	25.73 (14.01)	
		TT	842	86.9	30.26 (19.90)	
	rs28365085	TT	723	100.0	30.04 (19.27)	NA
	rs776746	AA	5	0.5	17.21 (9.72)	0.001
		AG	111	11.7	24.58 (14.12)	
		GG	832	87.8	30.34 (19.82)	
*SRD5A2*	rs11675297	AA	3	0.3	49.30 (11.95)	0.866
		AG	60	6.3	28.81 (22.57)	
		GG	888	93.4	29.61 (19.07)	
	rs2300700	AA	205	21.5	28.35 (18.27)	0.083
		AG	477	50.1	29.16 (19.92)	
		GG	271	28.4	31.70 (18.90)	
	rs4952197	AA	78	8.1	31.93 (17.42)	0.850
		AG	369	38.3	28.58 (18.31)	
		GG	517	53.6	30.06 (20.18)	
	rs523349	CC	394	41.8	30.16 (20.18)	0.790
	(V89L)	CG	466	49.5	28.85 (18.99)	
		GG	82	8.7	30.88 (17.17)	
	rs6732223	CC	240	26.0	32.16 (19.11)	0.104
		CT	468	50.8	28.28 (18.47)	
		TT	214	23.2	28.97 (19.15)	
	rs6760199	AA	77	8.0	32.56 (17.39)	0.920
		AC	380	39.3	28.75 (18.27)	
		CC	511	52.8	29.88 (20.19)	
	rs9282858	AG	64	8.9	28.09 (18.83)	0.630
	(A49T)	GG	659	91.1	30.27 (19.47)	
	rs9332975	AA	778	80.3	29.10 (19.35)	0.120
		AG	181	18.7	31.52 (18.82)	
		GG	10	1.0	33.19 (22.28)	
**Gene**	**SNP**	**Genotype**	**N**	**% Frequency**	**Finasteride (ng/mL)**	p-trend *
					**Mean (SD)**	
*SRD5A2L*	rs10001607	CC	292	30.6	29.15 (20.13)	0.950
*(SRD5A3)*		CT	466	48.8	30.00 (18.40)	
		TT	196	20.5	28.99 (18.67)	
	rs11133373	CC	449	46.4	29.62 (17.68)	1.000
		CG	433	44.7	29.73 (20.42)	
		GG	86	8.9	29.51 (21.41)	
	rs7663650	CC	400	41.3	30.23 (18.88)	0.480
		CT	465	48.0	28.98 (19.49)	
		TT	103	10.6	29.59 (19.98)	
	rs7682870	AA	588	60.9	29.72 (19.66)	0.890
		AC	336	34.8	29.07 (18.82)	
		CC	41	4.2	31.67 (16.25)	
	rs819270	AA	92	9.6	32.84 (22.29)	0.120
		AG	388	40.3	29.77 (18.17)	
		GG	483	50.2	28.81 (19.47)	

^#^ Treatment compliant men in the PCPT finasteride arm.

**Fig 1:**
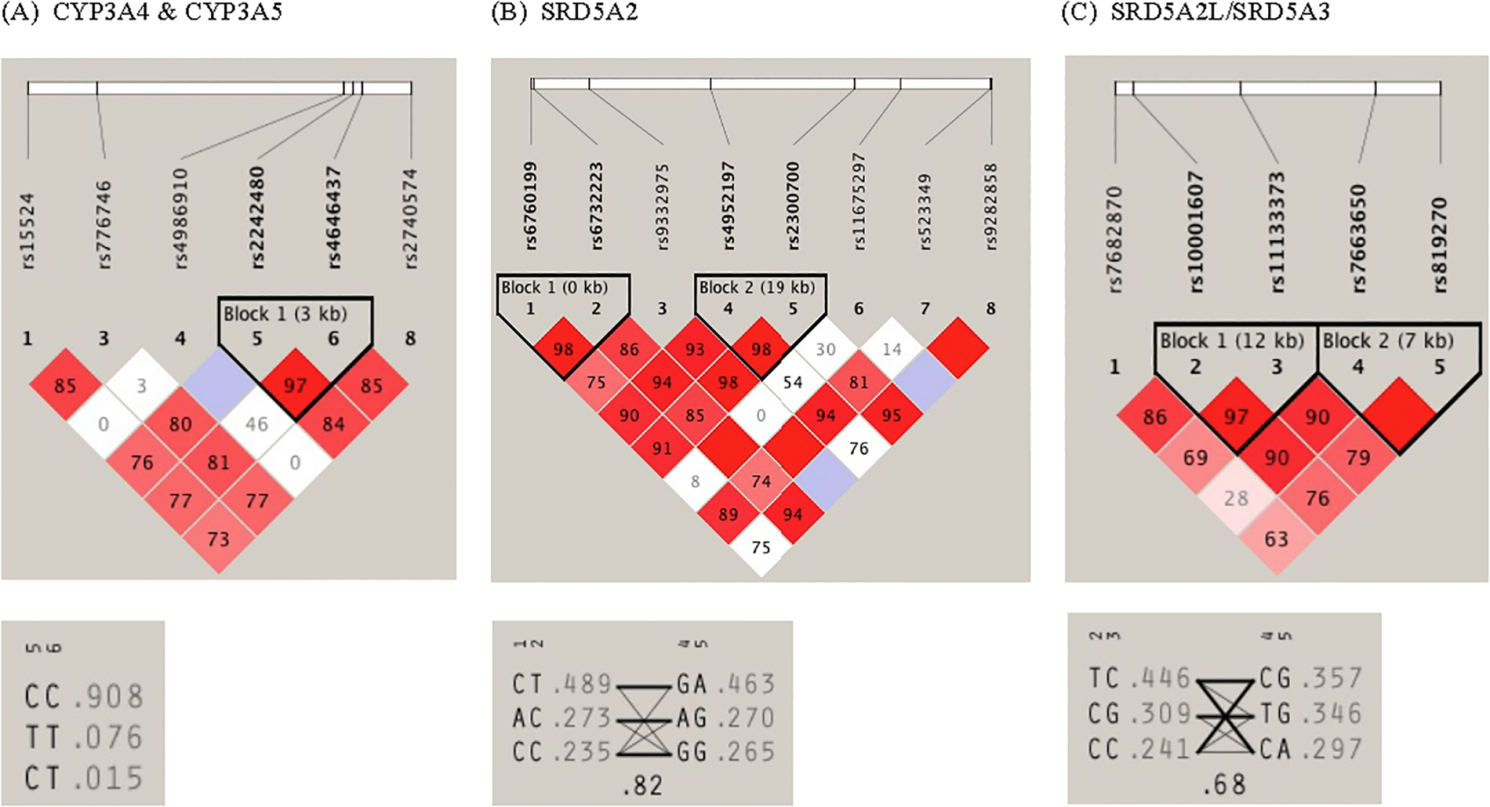
Linkage disequilibrium (LD) pattern and haplotype architecture for (A) *CYP3A4* & *CYP3A5*; (B) *SRD5A2*; and (C) *SRD5A2L/SRD5A3* genes. The haplotype block structure, as exhibited by Haploview is shown. LD was measured using data from all white subjects in the present study. The haplotype blocks were determined using the criteria described by Gabriel et al. The physical position of each SNP is presented in the upper diagram. Each diamond contains the level of LD measured by Hedrick's multiallelic D′ between pairs of single nucleotide polymorphisms. Shading shows the magnitude and significance of pairwise LD, with darker shades representing stronger LD; the diamond without a number corresponds to D′ = 1. Haplotypes for the variations and their population frequency (light gray color) are shown below each haplotype block of the corresponding genes. The SNP numbers across the top of the haplotypes correspond to those in the Haploview plot. D′ indicates the level of recombination between two blocks and is shown in the crossing area. The connection from one block to the next block is displayed through frequency corresponding to the thickness of the line.

The pairwise linkage disequilibrium (LD) structure was constructed with all SNPs evaluated for each gene. Since *CYP3A4* and *CYP3A5* are located adjacent to each other on chromosome 7, the SNPs in each gene were evaluated together, which revealed one LD block consisting of 2 *CYP3A4* variants (rs2242480 and rs4646437) (Fig 1A). A strong LD was observed between these two variants with haplotype frequencies of C/C 0.908, C/T 0.076, C/T 0.015. These two variants were also found to be associated with finasteride concentrations. For *SRD5A2*, two LD blocks were identified, with rs6760199 and rs6732223 in the same LD block (block 1) while rs4952197 and rs2300700 were in block 2 exhibiting high LD with corresponding haplotype frequencies shown in Fig 1B. For *SRD5A3*, two LD blocks were also identified with high LD (block 1: rs10001607 and rs11133373; block 2: rs7663650 and rs819270) as shown in Fig 1C.

## Discussion

The PCPT demonstrated that finasteride reduced the risk of prostate cancer by about 25% [15]. Based on these findings, we investigated whether finasteride concentrations may affect prostate cancer risk. In this nested case-control study from the PCPT, when we examined only individuals with detectable finasteride levels, we found no difference between any drug serum concentrations and the risk of overall, low- or high-grade prostate cancer. This suggests that since there was no concentration-dependent effect on prostate cancer, any exposure to finasteride intake may reduce prostate cancer risk. The observed effect could be attributed to participant adherence to therapy resulting in detectable drug levels, inter-individual variation in drug metabolism resulting in increased drug concentrations, or random variability from post-hoc analysis.

Moreover, we investigated the potential predictors of finasteride concentrations and found that drug serum concentrations were significantly associated with age at baseline and alcohol consumption specifically more than 30 g/d. Mean finasteride concentrations were higher in older participants, suggesting that perhaps the liver function (where finasteride is primarily metabolized) and metabolism of the older participants were more impaired compared to younger participants. We also found that men who consumed more than 30g/d of alcohol appeared to have higher drug concentrations. Results from a previous case-control study of the PCPT population demonstrated that heavy drinking (≥50g of alcohol consumption) made finasteride ineffective for reducing prostate cancer risk [20]. Whether alcohol consumption positively or negatively affects liver function and subsequent finasteride metabolism remains to be elucidated. Nonetheless, variability in the concentration may also be due to the time of day of blood draw as finasteride concentrations were lower for men who had their blood drawn later in the day (data not shown).

We next investigated whether polymorphisms in the enzyme target and metabolism genes of finasteride may in fact alter drug levels. Finasteride is a specific and potent steroid 5α-reductase type II inhibitor. Previous studies have demonstrated significant pharmacogenetic variation for finasteride at the *SRD5A2* locus where specific mutations (rs523349; rs9282858) affect enzyme stability or substrate binding by finasteride [11,12]. Our current genotyping study found no associations between finasteride concentrations and polymorphisms in enzyme targets of the *SRD5A2* or *SRD5A2L/SRD5A3* genes.

Genetic variants in the *CYP3A4* and *CYP3A5* genes involved in finasteride metabolism were significantly associated with serum concentrations in our study. The homozygous minor allele of *CYP3A4* rs2242480 and rs4646437 were associated with lower finasteride levels.

SNP rs2242480 (CYP3A4*1G) resides within intron 10 and reporter gene assays indicated that the minor allele has a significantly higher transcriptional activity [21]. Previous studies have demonstrated its association with lipid-lowering efficacy of atorvastatin [22] or tacrolimus pharmacokinetics in renal transplant patients [23]. SNP rs4646437 lies within intron 7 of *CYP3A4* and although the functional role of this SNP remains to be determined, it has been associated with CYP3A4 protein expression and enzyme activity in human liver microsomes observed in men only [24]. The variant allele in 3 SNPs (e.g. *CYP3A4* rs4986910; *CYP3A5* rs776746 and rs15524) were associated with higher finasteride concentrations. The minor allele of *CYP3A4* rs4986910 (CYP3A4*3), located in exon 12, significantly increased mean finasteride levels by more than 1.5-fold. While the allele frequency for this variant is low, studies have shown that the SNP was associated with reduced enzyme function, which may explain the higher finasteride levels observed [25]. SNP rs776746 (CYP3A5*3), located in intron 3 (6986A>G), produced a cryptic splice site and premature termination of the protein, resulting in loss of *CYP3A5* expression and subsequently affecting protein production and enzyme activity [14]. The wild type allele (CYP3A5*1) occurs at a lower frequency than the variant allele (CYP3A5*3). Homozygous carriers of the CYP3A5*3 variant allele produce very low or undetectable levels of functional CYP3A5 protein. On the contrary, heterozygous or homozygous carriers for CYP3A5*1 would display higher clearance and lower oral bioavailability of drugs, resulting in a lack of efficacy from a standard dose. Indeed, the homozygous AA genotype in rs776746 has been associated with poor response to imatinib therapy [26] and with decreased sunitinib response and tolerability [27]. Similar to finasteride, the rs776746 SNP has been association with serum tacrolimus concentration where carriers of a G/G genotype exhibited higher drug levels than the A allele (GA or AA) [28]. Moreover, higher mean finasteride concentrations have been found among carriers of the homozygous variant (T/T genotype) of *CYP3A5* rs15524 (CYP3A5*1D), which was similar to that observed for cyclosporine [28].

There are strengths and limitations to our study. The PCPT was a large placebo-controlled randomized trial that specified prostate cancer outcomes would be based on biopsy results. As such, the control group used in these analyses all had negative prostate biopsies, largely eliminating the possibility that controls may have had undiagnosed or undetected disease. Additionally, data were carefully collected throughout the course of the trial with a central pathology laboratory for uniform adjudication of all cases (including adjudication of Gleason grade) and we used a highly sensitive and specific assay for quantitating finasteride serum concentrations. However, our study was limited in that the PCPT included few minorities. Although we oversampled non-white controls to increase power for analyses by race, the power for any race-specific subgroups was hampered. Another limitation was that the finasteride concentrations were drawn from a very specific time-point, and hence don't provide any information about duration of use or long-term compliance on drug therapy. Given the drug’s short half-life, the low or undetectable levels of finasteride may have resulted from random missed dose(s) prior to sampling time or noncompliance. However, our analysis has shown that individuals with finasteride concentrations greater than 1 ng/mL correlated with their self-reported compliance of taking the drug therapy (data not shown). Finally, serum concentrations may not be representative of tissue levels.

## Conclusions

In summary, this study demonstrates the association between finasteride exposure and prostate cancer risk. Among treatment compliant men, there was no concentration-response effect of finasteride on disease risk. This is also the first study to show an association between finasteride concentrations and genetic variations in genes responsible for altering its metabolism pathway. We identified variants that influenced finasteride concentrations, which may explain the inter-individual variation observed in drug level differences. Our study has paved the way for future studies to conduct pharmacogenetic analyses of functional SNPs in finasteride-related metabolism genes that will likely contribute to an individual’s response to finasteride chemoprevention.
